# First Account of Phylogeographic Variation, Larval Characters, and Laboratory Rearing of the Endangered Cobblestone Tiger Beetle *Cicindelidia marginipennis*, Dejean, 1831 with Observations of Their Natural History

**DOI:** 10.3390/insects11100708

**Published:** 2020-10-16

**Authors:** Rodger A. Gwiazdowski, Jeremy C. Andersen, C. Barry Knisley, Brian P. Griffin, Joseph S. Elkinton

**Affiliations:** 1 Department of Environmental Conservation, University of Massachusetts, Amherst, MA 01002, USA; jcandersen@umass.edu (J.C.A.); brian.griffin@einsteinmed.org (B.P.G.); elkinton@umass.edu (J.S.E.); 2 Advanced BioConsulting, LLC., 139 Spring St., Shrewsbury, MA 01545, USA; 3 Department of Biology, Randolph-Macon College, Ashland, VA 23005, USA; bknisley@rmc.edu; 4 Department of Genetics, Albert Einstein College of Medicine, 1300 Morris Park Avenue Bronx, New York, NY 10461, USA

**Keywords:** *Cicindela*, *Cicindela marginipennis*, riparian, captive breeding, conservation genetics

## Abstract

**Simple Summary:**

Tiger beetles (Coleoptera: Cicindelidae) are highly predatory and colorful insects of long-standing fascination by entomologists. Most species in this group are in decline from range-wide habitat loss, including those with wide ranges such as the cobblestone tiger beetle (*Cicindelidia marginipennis* (Dejean, 1831)). This species is considered threatened and/or endangered range-wide, and conservation activities are hindered by a lack of basic information about this species’ biology. Here, we describe new aspects of this species’ basic biology in the lab and the field and use the mitochondrial locus cytochrome oxidase I (COI) to examine biogeographic patterns. We present larval descriptions with detailed drawings and find genetic evidence for geographically structured populations, suggesting that further conservation genetic research is warranted for this species. We expect the new tools and information presented in this paper will allow specialists to test further hypotheses about this species, advance survey methods, and guide proactive tiger beetle conservation.

**Abstract:**

The cobblestone tiger beetle, *Cicindelidia marginipennis* (Dejean, 1831) is a North American species specializing in riparian habitats from New Brunswick, Canada, to Alabama in the United States. In the United States, this species is state-listed as threatened or endangered range-wide and periodically receives consideration for federal listing, mostly due to habitat decline. Despite its conservation status, intraspecific genetic diversity for this species has not been explored and little is known about its natural history. To support further inquiry into the biology of *C. marginipennis*, this study provides the first look at range-wide genetic diversity using mitochondrial DNA (mtDNA), describes all three larval instars, and describes natural history characteristics from captive rearing and field observation. Based on mtDNA analyses, our results suggest that geographically based population structure may exist throughout the range, with individuals from Alabama possessing haplotypes not found elsewhere in our sampling. Further genetic analyses, particularly multi-locus analyses, are needed to determine whether the Alabama population represents a separate cryptic species. Our morphological analysis and descriptions of larval instars reveal a combination of characteristics that can be used to differentiate *C. marginipennis* from closely related and co-occurring species. Based on our field observations, we find that the larval “throw pile” of soil excavated from burrows is a key search image for locating larvae, and we provide descriptions and detailed photographs to aid surveys. Lastly, we find that this species can be successfully reared in captivity and provide guidelines to aid future recovery efforts.

## 1. Introduction

North American tiger beetles (Coleoptera: Cicindelidae) [1] are considered a flagship group for insect conservation because their colorful morphology helps identify them in the field and their vicious predatory behavior—using some of the best visual perception and response times known for any insect—makes them fascinating to observe [2,3,4,5]. Most species of tiger beetle specialize in dynamic habitats with sandy or clay-like soils including: deserts, dunes, grasslands, alpine ridges, riparian, and coastal areas. These open spaces allow adults and larvae to see prey and be seen by researchers, amateur naturalists, and photographers. This interest to see tiger beetles in the field is supported by a national field guide for the USA and Canada [6], and at least 10 regional guides [7]. These guides identify ~116 species and ~109 subspecies occurring in the US. and Canada; of which ~40% are habitat specialists, many of which are in decline largely due to habitat loss, and 16% (~36 species) that are currently rare enough to be considered for listing by the USA Fish and Wildlife Service [6,8].

Because tiger beetles can be the best-known organisms in their habitats, many species are important indicators of impacts to other biodiversity [8,9,10]. Consequently, in light of ongoing habitat loss and projected climate change [11], the taxonomy and phylogeny of many ostensibly well-known North American species and species complexes are being re-examined using genetic analyses e.g. [12,13,14]. Here, we follow the current tiger beetle taxonomy of Erwin and Pearson [15] that incorporates the important works of Rivalier e.g. [16,17] and Wiesner [18] and is prominently adopted by Pearson et al. [6] and Gough et al. [19] which updates the genus of *Cicindela marginipennis* to *Cicindelidia*.

The cobblestone tiger beetle, *Cicindelidia marginipennis* (Dejean, 1831) [20], is found in riparian areas on sandy cobblestone banks and bars (cobblestones are rounded stones ~5–8 cm [21] and larger) where seasonal flooding and/or ice scour enable within-river metapopulations to persist through dynamic flooding regimes that destroy and create habitat [22,23]. This species has an extensive (though disjunct) distribution for a tiger beetle; occurring in southern Alabama and Mississippi, absent throughout much of the southeastern USA, and occurring again through a northern corridor of states (Kentucky, Indiana, Ohio, West Virginia, Pennsylvania, New Jersey, New York, Massachusetts, Vermont, New Hampshire, and Maine) into New Brunswick, Canada [24]. Due to range-wide habitat loss, *C. marginipennis* is considered threatened and/or endangered throughout its range [25] and has periodically received federal consideration for listing as a threatened or endangered species since 1984 [26], most recently in 2019 [27]. This federal attention has produced the most comprehensive summary of this species’ conservation status [24]. This summary highlights that despite *C. marginipennis*’s extensive range, it remains under threat due to natural hydrological regimes and related effects of climate change, where further information about this species’ biology can help manage local extinction risks.

This study is based on a USA Fish and Wildlife Service sponsored project to explore genetic diversity and larval characteristics for *C. marginipennis* [28]. Here, we build on the results of this project by adding previously unpublished information about this species to report on: intra-specific genetic diversity from individuals sampled across the range of *C. marginipennis*; provide descriptions to identify all larval instars; and describe aspects of this species’ basic biology in the lab and the field. We expect the new information and tools presented in this paper will allow specialists to test further hypotheses about this species’ genetic diversity and ecology, advance survey methods, and guide proactive tiger beetle conservation.

## 2. Materials and Methods

### 2.1. Specimen Collection

Adult specimens of *C. marginipennis* were collected throughout this species’ range and stored in 95% ethanol for subsequent molecular analyses (see Table S1 for complete collection information). In addition, live specimens from the states of Vermont, New Hampshire, and a recently discovered locality in Maine [29] were collected and maintained in the laboratory to rear larvae for life-stage description (described below). Exact locations are not provided due to the endangered status of this species. Live specimens were collected under the following permits: State of Maine, Department of Inland Fisheries and Wildlife Permit # 2019-584; New Hampshire Fish and Game Department, letter of permission issued to the first author of 19 July, 2019. Vermont Agency of Natural Resources Authorization#: ER-2019-28.

### 2.2. DNA Extraction and Sequencing

Whole genomic DNA was extracted from single legs (preferentially a hind leg) of ethanol-preserved and laboratory specimens (post-mortem) using the Omega E.Z.N.A tissue DNA kit (Omega Bio-TEK, Norcross, GA, USA) following the manufacturer’s protocol. Fragments of the mitochondrial loci cytochrome oxidase I (COI) and cytochrome B (CYTB) were amplified using standard PCR protocols. For COI, PCR reactions were performed using two sets of primers. For all samples, we attempted to amplify fragments corresponding with the entire “barcode” region of COI using the primer pair LepF1 (5′-ATTCAACCAATCATAAAGATAT-3′) and LepR1 (5′-TAAACTTCTGGATGTCCAAAAA-3′) presented in Hebert et al. [30]. However, for many samples, these primers failed to produce amplicons. Therefore, the primer pair MLepF1 (5′-GCTTTCCCACGAATAAATAATA-3′) and HCO (5′-TAAACTTCAGGGTGTCCAAAGAATCA-3′) presented by Hajibabaei et al. [31] and Simon et al. [32], respectively, were utilized to amplify a fragment of the 3′ portion of the “barcode” region of COI. For CYTB, PCR reactions were performed using the primer pair CB1 (5′-TATGTACTACCATGAGGACAAATATC-3′) and CB2 (5′-ATTACACCTCCTAATTTATTAGGAAT-3′) presented by Crozier and Crozier [33]. For both loci, PCR reactions were performed in a 25 μL volume using Promega GoTaq (Promega, Madison, WI, USA) with 1 μL of eluted DNA (~2–5 ng/uL), 0.5 μL of each primer, 5 μL of Promega GoTaq 5× buffer, 0.5 μL of 10 μM dNTP mix (Promega, Madison, WI, United States), and 0.2 μL of Promega GoTaq *taq* polymerase. PCR products were then visualized on a 1% agarose gel, cleaned using Exo-SAP digestion, and sanger sequencing was performed on an ABI 3730 sequencer at the DNA Analysis Facility on Science Hill at Yale University (New Haven, CT, USA). Forward and reverse sequence reads were assembled and edited in Geneious v. R11 (BioMatters, Ltd., Auckland, New Zealand).

### 2.3. Sequence Alignment and Network Reconstruction

In addition to sequences generated in this study, we also obtained published COI sequences from the Barcode of Life Database: BOLD [34], via: http://www.boldsystems.org/ on a record search for “*Cicindela marginipennis*” as well as the following specimens representing the species: *C. sedecimpunctata* (NEON_BET_D14_000248), *C. highlandensis* (NEONTcarabid4098), *C. rufiventris* (NEONTcarabid4147 and NEONTcarabid4146), *C. schauppii* (NEONTcarabid4156), and *C. politula politula* (NEONTcarabid4144). Ingroup taxa include 10 specimens of *C. marginipennis* from BOLD, including 9 from New Brunswick, Canada, and one from Alabama (data presented in Table S1). For each locus, alignments were then constructed using MUSCLE [35], as implemented in Geneious R11 using default settings, and reading frames were examined to confirm that only mtDNA coding sequences were included. The COI alignment was then pruned to only include *C. marginipennis* individuals (no pruning was required for CYTB, as there were no published sequences in BOLD for this locus). Haplotype networks for each locus were then constructed based on the statistical parsimony method of Templeton et al. [36] using TCS v 1.21 [37] with a 95% confidence limit.

### 2.4. Phylogenetic Analysis of COI

Bayesian phylogenetic reconstructions were estimated for the full alignment (including outgroups) of COI using MRBAYES 3.2.7 [38,39], as implemented in the CIPRES Science Gateway [40]. A single-partition scheme was used, and MCMC searches were run for 10,000,000 generations, excluding a burn-in of the first 25% of generations, with default heating values. The phylogenetic reconstruction was visualized in FIGTREE Version 1.4.2. Uncorrected pairwise percent difference values were estimated by exporting a distance matrix of “percent identity” between all samples, as implemented in Geneious R11.

### 2.5. Laboratory Rearing of Cicindelidia marginipennis

Larvae were captive reared because field collecting *C. marginipennis* larvae for larval descriptions (below) is impractical for three reasons: (1) this species appears to have small local populations (fewer than 50 adults were observed during ideal field conditions at several isolated collection sites for this study) where substantial take may negatively impact that population; (2) larval burrows have been previously difficult to discover (see the above section); and (3) the burrow morphology and substrate in *C. marginipennis* habitat confounds conventional collection methods.

**Figure 1:**
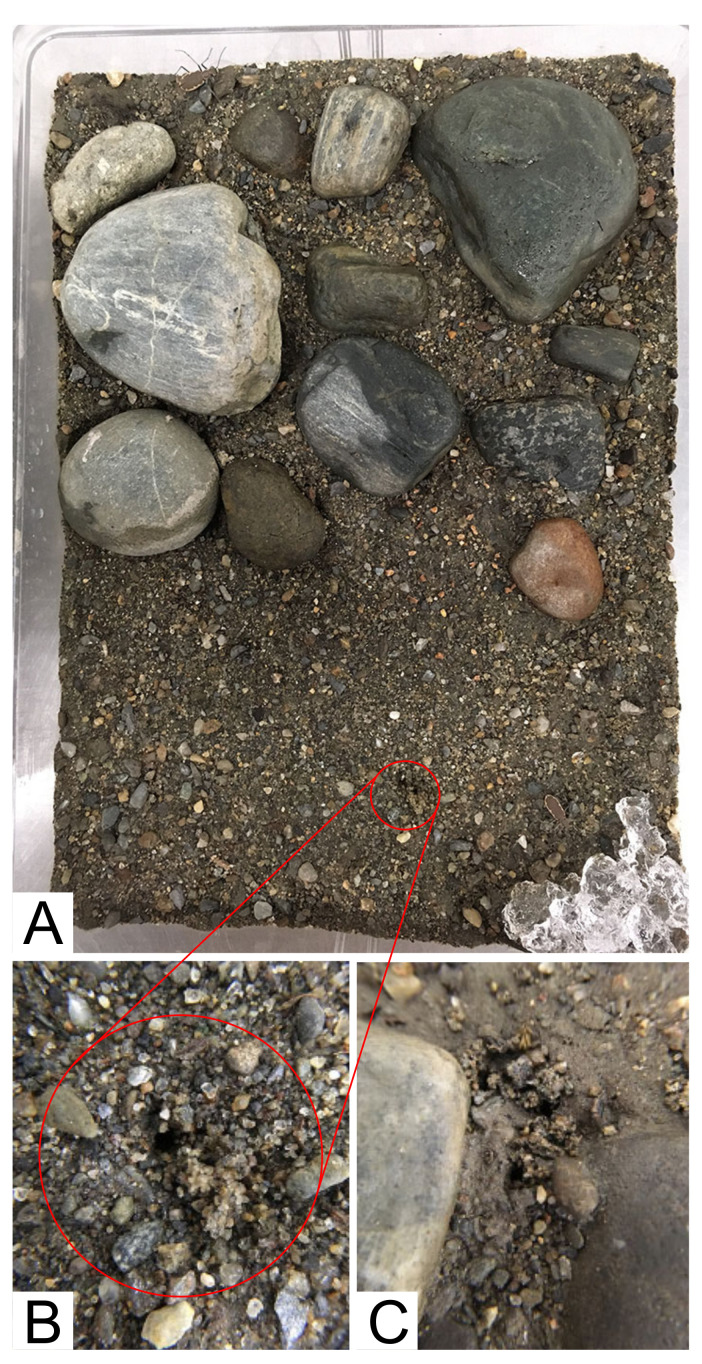
Overview of a rearing bin used in this study showing a mated pair of adults and the arrangement of cobbles and open area used for all bins, with detailed views of oviposition holes. (**A**) shows a view of the oviposition hole detailed in (**B**), (**C**) shows typical oviposition holes from a different bin, made at the base of, or adjacent to, cobbles.

Rearing methods followed those of Shelford [41] and Gwiazdowski et al. [42], which accommodate most North American species. *Cicindelidia marginipennis* adults were collected from the Connecticut River in Vermont and New Hampshire on 21 July, 2019, and from the Pemigewasset River in New Hampshire on 20 September, 2019 (methods below). Three forms of native soil were collected for rearing from the Vermont site: (1) small sized cobbles and pebbles; (2) substrate dug 0.3–0.6 m deep (sub-soil); and (3) substrate scraped from the top ~1 cm of the surface (top soil). This ~1 cm layer of top-soil is hypothesized to be what females encounter when ovipositing and often differs in composition from sub-soil by being finely grained [43]. Adults were collected using aerial nets and placed into a 19-dram translucent pop-top bottle including gel-water (described below) and shredded paper towel. Vials were held in a cooler to maintain temperature at ambient or slightly below. Adults were brought to the lab the same day as collected, with mated pairs placed in rearing bins (Figure 1A) lined with unbleached paper-towel substrate for 2 days, while the native soil was air-dried, then sanitized by baking in a convection oven at ~200 °C for 2+ hours. All laboratory equipment was regularly cleaned/sanitized using a solution of ethanol (95%), soap, and water (~1/2 tsp clear, scent-free dish soap; 350 mL ETOH; 600 mL H_2_O). Incipient growth of mold or fungus was controlled with 3% peroxide as recommended by Shelford [41]. Gel-water, a polyacrylamide gel, served as a source of free water when chewed by the beetles, mimicking their natural behavior of “chewing” moist soil to drink interstitial water. This gel was always present in a bin and was replaced regularly before desiccation. Food was available ad-libitum as ~1 week-old domestic crickets *Acheta domestica* (L., 1758) (Orthoptera: Gryllidae). Anecdotal observations from regular feeding of another tiger beetle species *Ellipsoptera puritana* (Horn, 1871) and our observations of *C. margninpennis* suggest that crickets of this age/size are fed upon more readily than either smaller or larger sizes [43].

**Figure 2:**
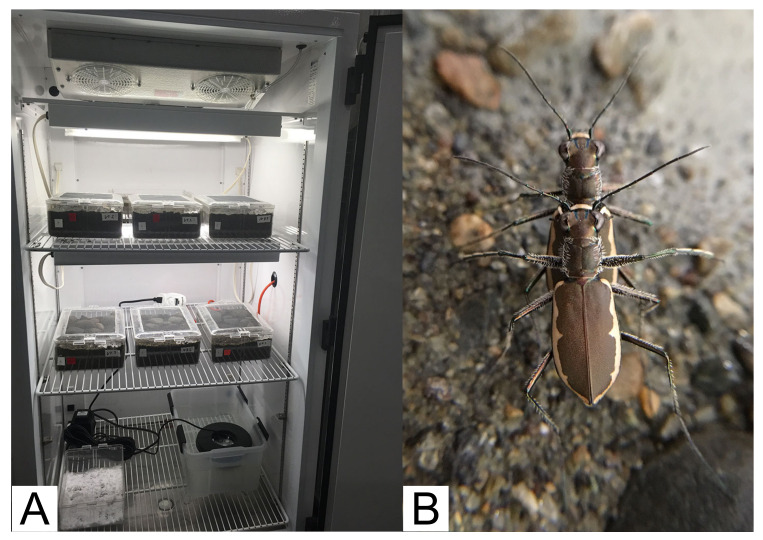
(**A**) shows the rearing bins arranged in the environmental chamber used for this study. (**B**) shows an overview of a coupled pair of *C. marginipennis* also from this study, with the male engaging in mate-guarding behavior, which often occurs after mating.

Rearing bins were set up with ~9 cm depth of dampened sub-soil, with an ~1 cm layer of top-soil, and cobbles pressed into the surface over half of the bin (Figure 1A). Females were kept in the same bin for the duration of rearing. As rearing began with pairs, after the death of any one male, males were rotated between bins daily, or semi-daily, only with females from their population. Bins were held in an environmental control chamber (Figure 2A) where lighting was provided by 20-Watt, 2 ft. 6500 K fluorescent tubes on a cycle of 14 h of light/10 h of dark, approximately corresponding to daylengths in Vermont during mid-July; temperature was on a 24 h cycle held at ~15–16 °C during the dark phase, then rising at the start of the light phase through a gradual arc to ~32–35 °C for three hours corresponding to ~11am through 2 pm then gradually cooling to reach ~15–16 °C at the start of the dark phase. Relative humidity was maintained at ~75–85%. Bins were removed from the chamber semi-daily (either morning or afternoon) for approximately 15 min for maintenance including feeding, cleaning, and misting the soil surface to replenish water, and to obscure oviposition holes/larval burrows, which were counted per bin (female) at the start of maintenance. Cobbles placed in bins were removed after the death of adults for that bin, allowing easier visualization of larval burrows for counting and feeding. Rearing in bins is efficient relative to staff time and the number of larvae produced but also makes it impossible to track development and mortality of individuals. This is because visualizing eggs requires skilled, labor-intensive removal of soil for careful sifting, and replacement of eggs (which can damage them), also larvae move/re-dig their burrows resulting in occasional cannibalism. Furthermore, individual larvae were periodically excavated for preservation, and these conditions make it impractical to critically examine survivorship.

### 2.6. Morphological Description of Larvae

Determining the identity of tiger beetle larvae is difficult, and rarely done for several reasons. While the adult stages are well known [6], only 65 of the ~116 North American species have been fully described [44,45]. Larvae of many USA species, including many that are widespread and common, have not been described, and the prevalent taxonomic key [46] is limited in scope, including species/specimens of uncertain identity. In addition, the characters used for identification require a microscope for determination, making field identifications challenging.

As larval tiger beetle descriptions are uncommon in the literature, we briefly review their history of development as an introduction to our methods. The first larval descriptions of North American tiger beetles are from Shelford [41] who provided partial descriptions of several larvae that occurred in the Chicago area. Subsequently, Hamilton [46] focused mostly on North American species, including several from Europe, and his descriptions standardized the morphological structures and terminology followed by subsequent workers. Hamilton [46] described 35 taxa including several subspecies and several specimens of unknown identity. Main sources and numbers of tiger beetle larval descriptions are Leffler [47] with 19 taxa (some were incomplete descriptions without figures); Knisley and Pearson [44] with 16, all from southeastern Arizona; Willis [48], 5 species; Gaumer [49], 5 subspecies of *Cicindela formosa*; Horn [50], 4 species; and 1 each by Beatty and Knisley [51], Kaulbers and Freitag [52], Spanton [53], and Willis [54]. Many of these descriptions, including most by Hamilton [46] and Leffler [47] were described from field-collected specimens, so the identity of some cannot be certain because multiple species occur in the same habitat.

**Figure 3:**
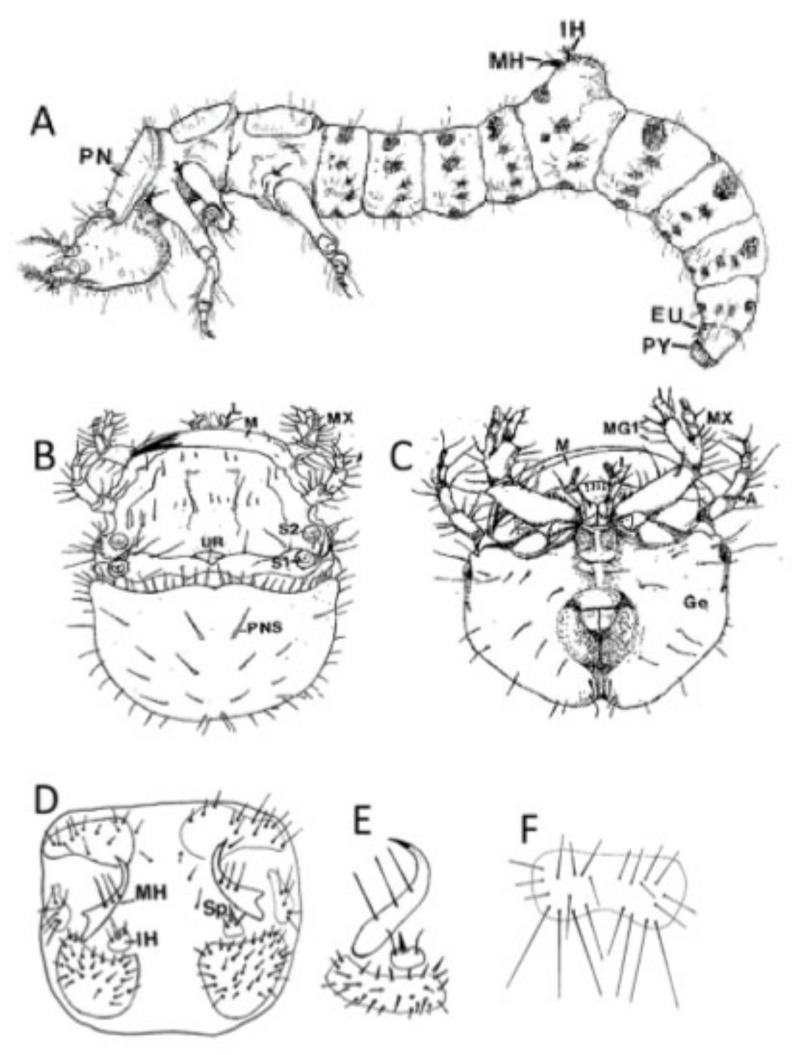
(**A**) Tiger beetle larva showing key characters: PN, pronotum; MN, median hooks; IH, inner hooks; EU, ninth eusternum; PY, pygopod. (**B**) Head, dorsal view, (**C**) ventral view: M, mandible; S1, stemmata I; S2, stemmata II; U, U-shaped ridge of frons; PNS, pronotal setae; MG1, galea of maxilla (for determining instar); MX, maxilla; A, antenna; Ge, gena; L, labium. (**D**) Dorsal view of fifth abdominal segment showing hooks Nin, sclerites, and setae. (**E**) Median and inner hooks and fifth caudal sclerite. (**F**) Ninth eusternum showing two groups of four setae.

Setal numbers are often the main identifying larval characters, including the number of setae on the pronotal disc, median, and inner hooks of the fifth abdominal segment, antennal segments, the ninth eusternum, and the pygopod of the terminal abdominal segment. There can be intra-specific variation in these numbers, so scoring multiple specimens is important to confirm identifications. Furthermore, many species share the same setal numbers within body regions and must be distinguished using a combination of characters. For example, nearly all species have either 2 or 3 setae on the median hooks, two setae on the inner hooks and either three or four pairs on the ninth eusternum. Hamilton [46] designated 12 pairs of setae on the pronotal disc of the first instar as primary setae (Figure 3A). Additional setae are added in later instars for some species. Determining the larval instar is also important, and fortunately, this is consistent and easy to determine using characters and relative size. First instars have one setae on the inner margin of the galea of the maxilla, second instars have two, and third instars have three (Figure 3C). Width of the pronotum increases through the three instars and can also be used to determine instar; this correlates with burrow diameter and is a useful character in field studies. Lastly, color of the pronotum and pronotal setae can be used to identify many species and, in some cases, can be easily distinguished in the field.

For the descriptions performed here, all larvae examined were confirmed to be *C. marginipennis*, because all three stages were reared in the laboratory (at the University of Massachusetts, Amherst) from field-collected adults obtained at one site in Vermont and two in New Hampshire (relative locations in Table S1). Several field-collected larvae from West Virginia (provided by Robert Acciavatti) matched the lab-reared specimens and were also used in the description. Rearing was conducted from September 2019 until early 2020. Representative larvae of first, second, and third instars were removed from their rearing chambers and preserved by immersing in boiling water for approximately one minute, then transferring to 80% ethanol for preservation, storage, and subsequent description [55]. Larvae from Vermont and New Hampshire were reared and analyzed separately, but there were no discernable differences between these populations (see results, below). The descriptions and the morphological features used and illustrated followed the approach of Knisley and Pearson [44] and Pineda and Kondratieff [56]. Material examined included at least five individuals of each of the three instars; variations among the characters used are included along with means for several of the key structures. Measurements were made under a Wild M3A dissecting microscope with an ocular micrometer and drawings made with a drawing tube attached to the microscope. All specimens used for description are held in the private collection of Dr. C. Barry Knisley.

## 3. Results

### 3.1. mtDNA Sequencing and Analyses

Thirty-two DNA barcode sequence fragments of COI of approximately 600 base pairs were recovered from individual specimens collected from the states of Alabama (*n* = 7), West Virginia (*n* = 5), New York (*n* = 4), Vermont (*n* = 3), New Hampshire (*n* = 11), and Maine (*n* = 2) often including different localities within a state. Twenty-one sequence-fragments of CYTB of approximately 419 base pairs were recovered from individual specimens collected from the states/province of Alabama (*n* = 5), West Virginia (*n* = 4), New York (*n* = 3), Vermont (*n* = 3), New Hampshire (*n* = 4), and Maine (*n* = 2). Not enough specimens had sequences from both COI and CYTB for a combined analysis (see Section 2.2, above), and so each locus was analyzed separately. All specimens are held at −20 °C in the Elkinton Lab, at the University of Massachusetts, Amherst. All sequences have been deposited in GenBank under a continuous series of accession numbers: COI; MT904220-MT904251, CYTB; MT904252-MT904272. Accession numbers, including GenBank numbers, along with all locality and ancillary information for all specimens are provided in Table S1.

### 3.2. Phylogeographic Diversity

**Figure 4:**
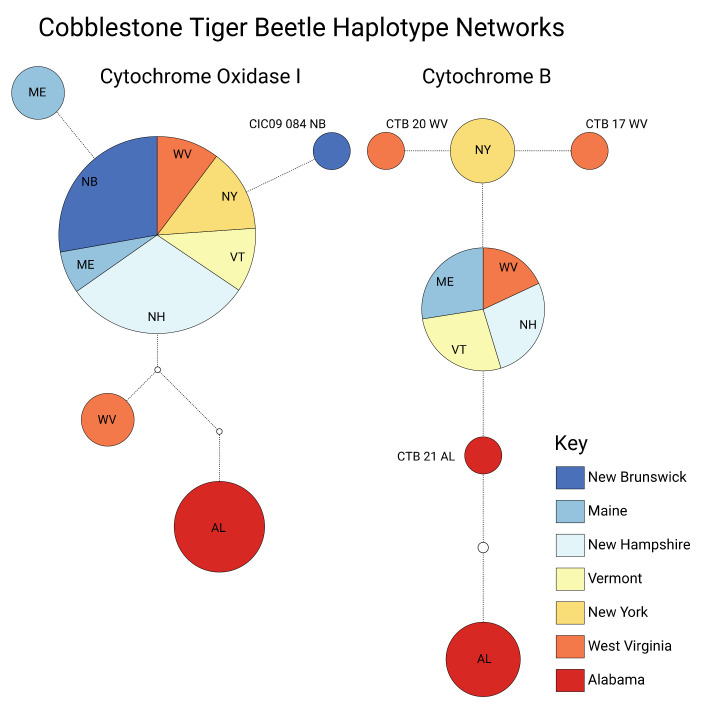
Haplotype networks obtained from the TCS analysis for cytochrome oxidase I (COI) and cytochrome B (CYTB). The size of the circle represents the frequency of each haplotype. Numbers next to small colored circles indicate the individual specimen for that unique circle/haplotype. Empty circles represent hypothetical haplotypes not observed in this study. For the COI network, Clade B comprises only specimens from Alabama, whereas Clade A comprises all other specimens/localities sampled.

**Figure 5:**
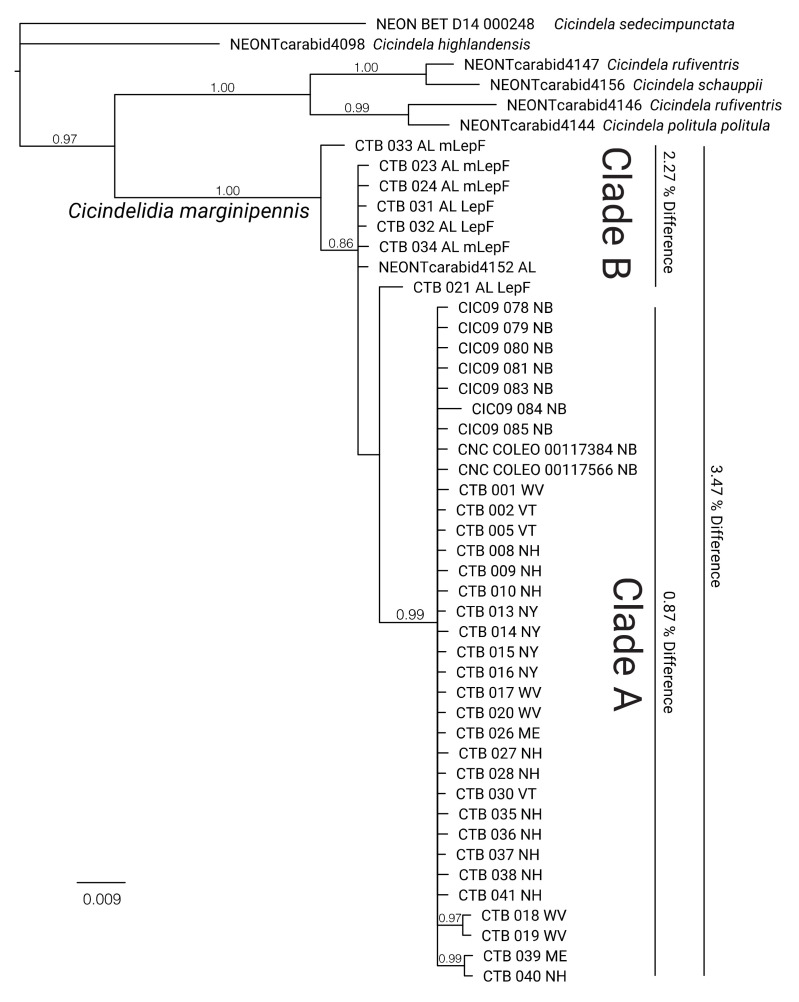
COI Bayesian genealogy showing accession numbers and abbreviations for state/province for all *C. marginipennis* specimens in this study, rooted using closely related species. Clades A and B show specimens that directly correspond to the same clades indicated in the COI haplotype network of Figure 3. Percent % differences shown on the small inner bars are calculated from within those clades. Percent difference on the large outer bar is calculated between Clades A and B. Posterior support values for each node appear at the base of that node. The scale bar represents estimated substitutions per site.

The population structure within our sampling corresponds to a known geographic division of population occurrence for this species [24], where all COI haplotypes of Clade B are from Alabama, and all other haplotypes are from the Northeast, including West Virginia (Figure 4 and Figure 5). This pattern also directly agrees with the haplotype structure based on CYTB (Figure 4). Clade A is comprised of the most-common haplotype. Within Clade A, both loci show several unique haplotypes, with a calculated ~0.9% intra-clade difference at COI (Figure 5).

### 3.3. Laboratory Rearing

*Cicindelidia marginipennis* individuals were reared in the lab for the first time to generate and prepare specimens for formal description and notes below describe the rearing process, main observations, and comparative aspects relative to other species. Field collection of adults and laboratory rearing of larvae provided incidental insights for identifying larval burrows in the field, and this is discussed in Section 4.3, below.

A timeline of main rearing events is presented in Table 1. Adults appeared to behave normally where feeding, mating, and mate guarding was frequently observed (Figure 2B). Life spans of unknown-age adults brought into the lab were consistent with or considerably extended from what would be expected in the wild (Table 2). Mortality always appeared to occur rapidly, with beetles behaving normally (feeding, ovipositing, mating, etc.) then presenting as moribund or dead ~24 h later. *C. marginipennis* oviposition holes were regularly observed in the lab during maintenance (Figure 1A,B) and appeared to be frequently placed at the edge or base of cobbles and pebbles (Figure 1C), which is consistent with Leonard and Bell’s [22] report of them “in open sandy areas between cobblestones”.

Total counts of oviposition holes roughly corresponded to the maximum number of active first instar larval burrows recorded on any one day (Table 2); here, this maximum of active burrows is considered a proxy for female fecundity and is likely a conservative estimate. Hatch appeared to occur ~20 days after egg laying. Notably, development from 1st to 3rd instar was longer than expected for *C. marginipennis* (Table 1). Most larvae remained in first instar for 5–6 months, despite constant environmental conditions and food availability. During this time, larvae appeared to plug their burrow and move down within it, remaining stationary for several weeks where they could be observed through the bottom of the clear bin. The authors have never observed this prolonged inactivity of first instars of other species they have reared. A brief experimental rise in temperature for a subset of bins given a dark phase low of 24 °C over the course of three weeks did not appear to stimulate activity. Subsequently, all remaining larvae from each state (Vermont and New Hampshire ~40 larvae) were, respectively, transferred into two 10-gallon glass aquaria filled with ~20 cm of sub-soil, lit with single 40 W incandescent reptile lights (Exo-Terra, Hagen, Montreal, QC, Canada) with the same photoperiod, a dark phase low temperature of 24 °C and a constant light phase high of ~29–30 °C. In these conditions, within 2–2.5 months, almost all larvae had transitioned to the 3rd instar, and more frequent feeding could have shortened this time. Lastly, *C. marginipennis* larvae of all life stages appear to react more sensitively to common disturbances than other species of North American tiger beetles reared in the lab. Larvae appear more apt to drop down in their burrow when sensing large movements (i.e., people or shadows) from greater distances and from slighter vibrations than appear to trigger similar species. They also, anecdotally, appear to remain down in their burrow longer (~15–30 min or more) than similar species [57].

### 3.4. Larval Descriptions

#### 3.4.1. First (1st) Instar Larva

Color: head and labrum black; antennae dark brown to black; mandibles with basal portion dark brown, distal portion dark brown to black; labium brown; pronotal setae white, glassy, other body setae yellow; pronotum black; mesonotum and metanotum light brown; abdominal sclerites light brown, slightly darker than membranous areas.

Head: dorsal setae prominent; diameter of stemmata 1 distinctly larger than stemmata II; U-shaped ridge on frons with no setae; antennal segment 1 (basal segment) with no setae, segment 2 with 2–3 setae; segment 3 with 3, rarely 2 setae; segment 4 with 3 setae.

**Figure 6:**
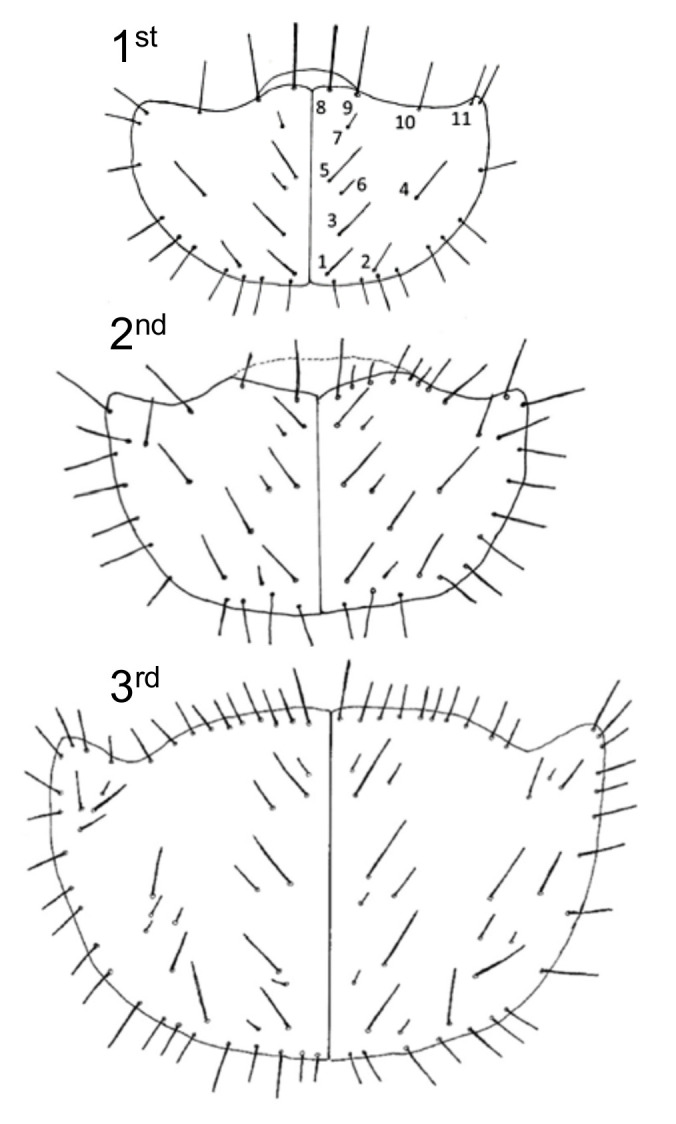
Generalized images of pronotal discs of *C. marginipennis*, derived from larvae observed in this study, showing setal numbers and arrangement. Top, first instar; middle, second instar; bottom, third instar; all shown in relative size—not to scale.

Thorax: pronotum broad with lateral margins extended nearly as far anteriorly as the medial portion; pronotal disc with 12–15 setae, several short to minute, none on cephalolateral angles (Figure 6).

Abdomen: Sclerotized areas only slightly darker than membranous portions; both tergites of the third abdominal segment with 4–6 setae; eusternum of ninth abdominal segment with 2 groups of 3 setae; pygopod with 5–6 setae on each side; median hooks of fifth abdominal segment with 2 setae on the middle third of the hook; inner hooks of fifth abdominal segment with 2 setae and the spine about equal to or greater than one-half the length of the entire hook.

Measurements (means of five specimens): Total length, 7.2 mm; width at third abdominal segment, 0.83 mm; diameter of stemmata 1, 0.13 mm; diameter of stemmata II, 0.08 mm; distance between stemmata I and II, 0.08 mm; pronotal width 1.82 mm; pronotal length 0.90 mm; length of antennal segment 1, 0.08 mm; segment 2, 0.14 mm; segment 3, 0.11 mm, fourth segment, 0.096 mm.

#### 3.4.2. Second (2nd) Instar Larva

Color: Head and labrum black, shiny; antennae dark brown; mandibles with basal portion light brown, distal portion black; labium brown; pronotal setae white, glassy, other body setae yellow; mesonotum and metanotum brown; abdominal sclerites light brown.

Head: Dorsal setae prominent; diameter of stemmata 1 distinctly larger than stemmata II; U-shaped ridge on frons with 2 setae; antennal segment 1 with 4–5 setae, segment 2 with 5, rarely 6 setae; segment 3 with 2 setae; segment 4 with 3 setae.

Thorax: Pronotum broad with lateral margins extended nearly as far forward as the medial portion; pronotal disc with 20–23 setae, some small to minute, cephalolateral angles with 1–2 setae (Figure 6).

Abdomen: Sclerotized areas distinct; both tergites of third abdominal segment with 6–7 setae; eusternum of ninth abdominal segment with 2 groups of 4, rarely 3 setae; pygopod with 7–8 setae on each side; median hooks of fifth abdominal segment with 2 setae on the middle third of the hook; inner hooks of fifth abdominal segment with 2 setae and the spine one-half or greater than the length of the entire hook including spine.

Measurements (means of five specimens): Total length, 9.9 mm; width at third abdominal segment, 0.95 mm; diameter of stemmata 1, 0.18 mm; diameter of stemmata II, 0.13 mm; distance between stemmata I and II, 0.12 mm; pronotal width 1.37 mm; pronotal length 0.98 mm; ratio of pronotal length/width, 0.72 mm; length of antennal segment 1, 0.19 mm; segment 2, 0.22 mm; segment 3, 0.14 mm, segment 4, 0.128 mm.

#### 3.4.3. Third (3rd) Instar Larva

Color: Head and labrum black, shiny; antennae dark brown; mandibles brown basally, distal half dark brown to black; labium brown; pronotal setae white, glassy, other body setae yellow; mesonotum medium brown; metanotum light brown; abdominal sclerites light brown.

Head: Dorsal setae prominent; diameter of stemmata 1 distinctly smaller than stemmata II; U-shaped ridge on frons with 2 setae; antennal segment 1 with 3–4 setae, segment 2 with 6–8 setae; segment 3 with 2 setae; segment 3, rarely 4 setae.

Thorax: Pronotum broad with lateral margins extending as far forward as the medial portion; pronotal disc with 26–38 setae, including as many as 14 that are small to minute (Figure 6); usually 2 but sometimes 4 setae on the cephalolateral angles.

Abdomen: Sclerotized areas distinct; both tergites of the third abdominal segment with 11 setae; eusternum of ninth abdominal segment with 2 groups of 4 setae (Figure 3F); pygopod with 8, rarely 7 or 9 setae on each side; median hooks of fifth abdominal segment with 3 setae, all on the middle third of the hook (Figure 3E); inner hooks of fifth abdominal segment with 2 setae and the spine greater than one-half the length of the entire hook (Figure 3E).

Measurements (mean of five specimens): Total length, 17 mm; width at third abdominal segment. 1.44 mm; diameter of stemmata 1, 0.27 mm; diameter of stemmata II, 0.21 mm; distance between stemmata I and II, 0.19 mm; pronotal width 2.10 mm; pronotal length 1.38 mm; ratio of pronotal length/width, 0.66 mm; length of basal antennal segment 1, 0.34 mm; segment 2, 0.32 mm; segment 3, 0.26 mm; segment 4, 0.22 mm.

#### 3.4.4. Key Characters for Distinguishing 3rd Instar *Cicindelidia marginipennis*

Important third instar characters for *Cicindelidia marginipennis* include:

1. Head and pronotum black;
2. Twenty-six to 38 setae on the pronotal disc (but many small to minute);
3. Inner hooks with spine about one-half or greater than total length of the spine;
4. Four pairs of setae on 9th eusternum;
5. Three setae on each medial hook.

While all of these characters are common to many species, the combination of them is common to only a few of the USA species. In particular, the combination of four setae on the paired ninth eusternum and three setae on each median hook is shared by only a few described species, none co-occurring with *C. marginipennis*. If we follow the taxonomic key to third instar larvae of Northeastern USA tiger beetles by Leonard and Bell [22], *C. marginipennis* ends at couplet 14, also ending at *Cicindela tranquebarica* (Herbst, 1806), which has a bright coppery bronze head and pronotum—not black like *C. marginipennis*. For field studies with *C. marginipennis*, it is significant that the only two other tiger beetle species likely to co-occur in the same habitat are *Cicindela repanda* (Dejean, 1825) and *Cicindela ancocisconensis* (Harris, 1852). Third instars of *C. repanda* have only one setae (rarely an additional small one) on the median hooks and a dark coppery bronze head and pronotum. *C. ancocisconensis* has four, not three, setae on the median hooks and three not four groups of setae on the ninth eusternum.

## 4. Discussion

### 4.1. Cryptic Diversity within C. Marginipennis

Recent, revelatory, taxonomic work in divergent groups of North American tiger beetles indicates range-wide genetic analysis is imperative for understanding diversity in this taxon. For example, several cryptic species of tiger beetles have been recently described, and more are hypothesized to occur among the North American fauna [13]. Conversely, several well-known, phenotypically distinct, tiger beetle subspecies are not supported as distinct lineages [14].

In this study, specimens collected from Alabama form a monophyletic group (Figure 5, Clade B), and pairwise mtDNA (COI) difference between these specimens and all other *C. marginipennis* is ~3.5 % (Table 3), which is consistent with species-level mtDNA differences in other North American tiger beetles [58,59]. Most of the specimens in the COI haplotype network comprise one most-common haplotype (Figure 4), which is a pattern typical of many insect species occupying large ranges e.g. [60,61,62,63]. Within Clade A of Figure 5, both loci show several unique haplotypes, with a calculated ~0.9% intra-clade difference at COI (Figure 5). This intra-clade difference, combined with the additional haplotypes observed in CYTB, suggests additional mtDNA sampling of more individuals and localities and/or loci with finer resolution (e.g., SNPs or microsatellites) may reveal additional population structure at regional scales. This possibility is highlighted by the haplotype diversity seen across both loci in samples from West Virginia (Figure 5 and Figure 6). In particular, the mtDNA monophyly and amount of mtDNA difference observed in specimens from southern Alabama, together with the known disjunction from their nearest populations in Kentucky, suggest Alabama populations are deserving of further taxonomic investigation regarding their species status. Overall, the results from this first study of genetic variation in *C. marginipennis* suggest further undiscovered diversity may be present throughout this species’ range. Future taxonomic investigation of this group should include range-wide sampling that represents most known localities and major river systems, more intra-population sampling from Alabama and re-exploration of historic sites in Mississippi, or use of museum specimens from those localities. Characterization of the Alabama populations relative to their potential species status may be best addressed with complimentary analyses using unlinked molecular markers (i.e., mitochondrial and nuclear loci) morphological variation, and possibly ecological differences.

### 4.2. Future Recommendations for Rearing C. marginipennis

Most North American tiger beetles can be reared in the laboratory as outlined by Gwiazdowski et al. [42] with consideration for species-specific conditions [64]. This first rearing of *C. marginipennis* showed results comparable to similar species discussed by Gwiazdowski et al. [42]. Notably, oviposition, or signs thereof, was never seen during the day and presumably takes place during night or early morning as observed with the Puritan tiger beetle [65]. In addition, no obvious signs of fungi, disease, or parasites were observed, which presents a real threat for rearing rare invertebrates with small population sizes [66]. Mites were not detected either (presumably phoretic), which have been observed on related species *E. puritana* in the Chesapeake bay, and the Northeastern Beach tiger beetle (*Habroscelimorpha dorsalis dorsalis* Say, 1817) in New England [67].

The results of this study suggest rearing for *C. marginipennis* could be scaled to support reintroduction efforts for unoccupied or newly created habitats [68]. For rearing large numbers of tiger beetle larvae in a single season, it may be best to capture adults as early as they emerge. However, for this study, adult *C. marginipennis* collected on September 20, 2019, mated normally and produced a useful number of larvae (Table 2). This September 20 collection date is past a generally presumed senescence of adults in late August and early September. Future rearing projects that draw adults from small, or isolated populations, could consider proceeding in the laboratory over the winter months using adults collected later in the flight season. Notably, changes in two rearing conditions may contribute to greater fecundity and faster development: (1) increasing the soil temperature for eggs and (2) increasing the soil depth for larvae.

Hatch of *C. marginipennis* eggs occurred ~20 days after laying. This ~20-day duration is consistent with lab rearing, under similar conditions, for *E. puritana* [69]. However, this is inconsistent with the ~2–10 days observed for other species [42]. It is possible time-to-hatch for *C. marginipennis* and *E. puritana* may be an artifact from cooler-than-natural soil temperatures in the lab. Field observations of *E. puritana* oviposition behavior by the first author in 2019 and 2020, during ideal conditions in the breeding season, recorded temperatures directly adjacent to *E. puritana* oviposition holes of ~24 °C at night and >38 °C during the day [65]. These field conditions are likely similar for *C. marginipennis*. This is a much warmer range for eggs than used here for rearing *C. marginipennis* and for *E. puritana* [69]. Warmer laboratory temperatures for eggs may bring hatch into the 2–10 days interval [42], and future rearing attempts for this and similar species should consider hatching eggs under a different temperature regime than adults. Lastly, the relatively rapid larval development that occurred subsequent to their placement in deeper soil may suggest a species-specific requirement, and future rearing efforts for this species should account for this hypothesis.

### 4.3. Finding Larvae in the Field

**Figure 7:**
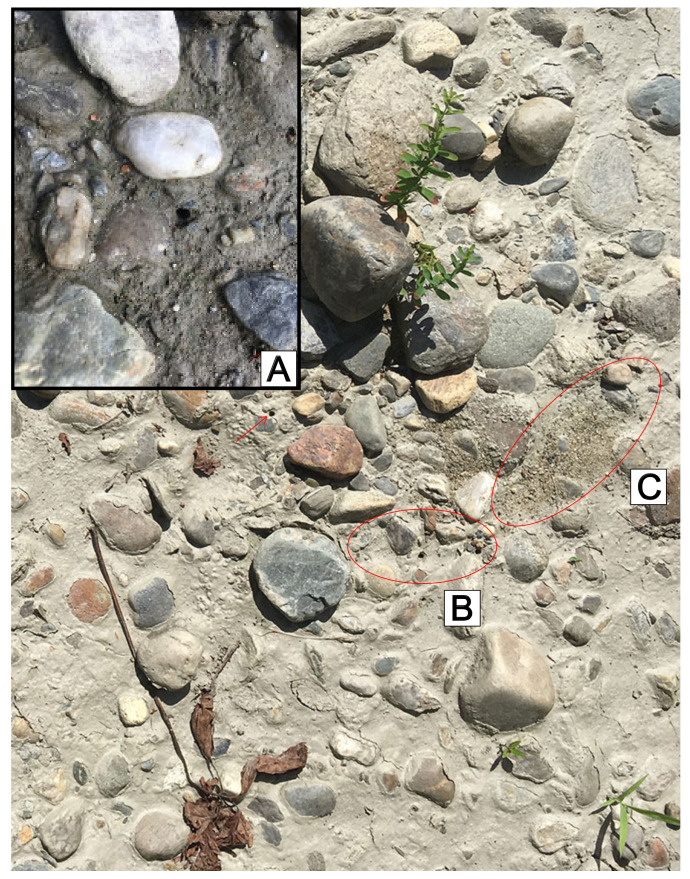
*C. marginipennis* larval burrows are difficult to detect amid heterogenous cobble habitats. Burrow mouths can blend in with shadows, with substrate and occur at acute angles as in (**A**). Visually searching for a “throw pile” of soil excavated and ejected from larval burrows is easier to detect for this species (**C**), which can be connected to one or several adjacent burrows (**B**). Following the visual cue of a “throw pile” (**C**) can lead to detecting one or several burrows (**B**). Close investigation of the area in B reveals three adjacent burrows, and a fourth burrow at the upper left.

Most species of North American tiger beetle larvae create burrow mouths (top opening of the burrow) roughly perpendicular to a vertical burrow, appearing as round black holes on the surface of relatively open areas of flat, sandy, or clay-like substrate [55]. *C. marginipennis* larval burrows have been notoriously difficult to find [70,71], because they do not present as most other species and are initially difficult to visualize in the field for a couple reasons: (1) cobbles create a complex, heterogenous background including shadows which obscure burrows; (2) larval burrow mouths are often made under the base of cobbles and are frequently angled relative to the surface, sometimes at angles of ~90 degrees (Figure 7A). These characteristics confound the conventional search image for larval burrows, so a key solution is to search for the “throw pile” of soil larvae excavate from their burrow (Figure 7C). Similar to other North American species, *C. marginipennis* larvae unidirectionally toss substrate away from the burrow mouth (Figure 7B). This behavior creates an oval, or cone-shaped pile of soil, which will appear dissimilar to, and visibly distinct from, the surrounding surface substrate (Figure 7C) because the pile’s soil is either: (A) darker than the surface from sub-surface moisture; (B) lighter from desiccation; or (C) different in size and composition from the surface soil. The throw pile can be obscured by recent or active rain. However, larvae appear to re-open or clean their burrow after rain in the field, or wetting in the lab, which creates throw pile of a distinctly different color than the surrounding surface. In arid conditions, the difference in soil texture is visually useful (Figure 7). This search image can be effectively used to guide surveys for *C. marginipennis* larval burrows with at least two caveats: (1) casual observations likely underestimate larval density because burrows without throw piles may be overlooked and are otherwise visually obscured, and larvae may have plugged burrows (as the case with many species), so systematic surveys (e.g., using quadrats, etc.) may be more reliable for density estimates; (2) *C. marginipennis* may not be the only species in this habitat, as other species with angled larval burrows may co-occur such as *C. repanda*, although adult *C. repanda* are observed in slightly different microhabitats than the cobble areas occupied by *C. marginipennis* [71,72]. Monitoring methods have been developed for adult *C. marginipennis* by Hudgins et al. [73,74], and using the larval throw pile as a cue for detecting *C. marginipennis* burrows could assist development of larval monitoring methods or as a cue when exploring newly identified habitats [75].

*Cicindelidia marginipennis* larval burrows appear to be twisted or curved throughout their depth and occur in heterogenous rocky substrate of densely packed sand and clay. Larvae of many other North American species can be easily recovered using a straight probe to guide excavation of loose soil or by patiently positioning a trowel adjacent to the burrow mouth and waiting several minutes for the larvae to return to the surface, then stabbing underneath it with the trowel, blocking the larvae near the surface for easy collection. Neither of these methods seems practical with *C. marginipennis*, and laboratory rearing ensured the described larvae were from the identified adults.

## 5. Conclusions

The cobblestone tiger beetle, *Cicindelidia marginipennis*, a North American riparian species, occurs from Alabama in the United States through New Brunswick, Canada. This species is endangered throughout its range due to habitat loss, and this study presents several new lines of information about its biology that are of direct relevance to its future study and conservation. First, a range-wide mtDNA survey finds the possibility of a cryptic species in Alabama, and further population structure throughout the range; second, all larval instars are formally described with a key provided for their identification; third, visualizing the larval “throw pile” of soil excavated from their burrows is a key search image for locating these larvae in the field; lastly, results from captive rearing provide basic information about this species’ life history and indicate it can be lab-reared for conservation efforts.

## Figures and Tables

**Table 1:** Dates and main events in the course of rearing all instars of the cobblestone tiger beetle.

Date	Rearing Event
21 July 2019	Adults field collected and brought into the lab
23 July 2019	Adults moved into oviposition bins with native soil
4 August 2019	First oviposition
26 August 2019	First hatch (1st instar)
26 September 2019	First 2nd instar
6 January 2020	Moved to larger aquaria
22 January 2020	First 3rd instar
30 March 2020	Most remaining larvae in 3rd instar

**Table 2:** Survival and estimated fecundity of cobblestone tiger beetle adults used for laboratory rearing of larvae, collected in summer/fall of 2020.

Max. 1st Instar	Sex	Days Alive	Collected	Deceased	Accession #
34	Female	54	21 July	13 September	VT1
32	Female	75	21 July	4 October	NH1
11	Female	36	21 July	26 August	VT3
10	Female	33	21 July	23 August	NH3
9	Female	31	21 July	21 August	NH2
8	Female	44	21 July	3 September	VT2
20	Female	19	20 September	9 October	NH5.1
5	Female	14	20 September	4 October	NH4
3	Female	8	20 September	28 September	NH5
N/A	Male	70	21 July	29 September	NH3
N/A	Male	69	21 July	28 September	NH1
N/A	Male	54	21 July	13 September	VT1
N/A	Male	38	21 July	28 August	VT3
N/A	Male	36	21 July	26 August	VT2
N/A	Male	8	20 September	28 September	NH4

**Table 3:** Pairwise percent difference values for COI for all specimens in this study, estimated by exporting a distance matrix of “% identity” between all samples, as implemented in Geneious R11. Values below the diagonal represent average pairwise percent differences between regions, values along the diagonal represent average pairwise differences within regions, and values above the diagonal represent the maximum pairwise percent difference between individuals in each region.

Region	Alabama	ME/NH	Northeast	WV
**Alabama**	99.31	3.47	3.47	3.20
**ME/NH**	1.72	100.00	0.58	0.66
**Northeast**	1.50	0.33	99.94	0.87
**WV**	1.45	0.65	0.49	100.00

## Supplementary Materials

The following are available online at https://www.mdpi.com/2075-4450/11/10/708/s1, Table S1: CTB Specimen Supplementary Table S1.

## References

[1] Duran D.P., Gough H.M. (2020). Validation of tiger beetles as distinct family (Coleoptera: Cicindelidae), review and reclassification of tribal relationships. Syst. Èntomol..

[2] Mizutani A., Toh Y. (1998). Behavioral analysis of two distinct visual responses in the larva of the tiger beetle (*Cicindela chinensis*). J. Comp. Physiol. A.

[3] Pearson D.L., Vogler A.P. (2001). Tiger Beetles.

[4] Toh Y., Okamura J.Y. (2001). Behavioural responses of the tiger beetle larva to moving objects: Role of binocular and monocular vision. J. Exp. Biol..

[5] New T.R. (2010). Beetles in Conservation.

[6] Pearson D.L., Knisley C.B., Duran D.P., Kazilek C.J. (2015). A Field Guide to the Tiger Beetles of the United States and Canada: Identification, Natural History and Distribution of the Cicindelidae.

[7] Pearson D.L., Shetterly J.A. (2006). How Do Published Field Guides Influence Interactions between Amateurs and Professionals in Entomology?. Am. Èntomol..

[8] Pearson D.L., Cassola F. (1992). World-Wide Species Richness Patterns of Tiger Beetles (Coleoptera: Cicindelidae): Indicator Taxon for Biodiversity and Conservation Studies. Conserv. Biol..

[9] Pearson D.L., Carroll S.S. (1998). Global Patterns of Species Richness: Spatial Models for Conservation Planning Using Bioindicator and Precipitation Data. Conserv. Biol..

[10] Knisley C.B. (2011). Anthropogenic disturbances and rare tiger beetle habitats: Benefits, risks, and implications for conservation. Terr. Arthropod Rev..

[11] Wuebbles D., Easterling D., Hayhoe K., Knutson T., Kopp R., Kossin J.P., Kunkel K., LeGrande A., Mears C., Sweet W., Wuebbles D.J., Fahey D.W., Hibbard K.A., Dokken D.J., Stewart B.C., Maycock T.K. (2017). Our Globally Changing Climate. Climate Science Special Report: Fourth National Climate Assessment.

[12] Knisley C.B., Kippenhan M., Brzoska D. (2014). Conservation status of United States tiger beetles. Terr. Arthropod Rev..

[13] Duran D.P., Herrmann D.P., Roman S.J., Gwiazdowski R.A., Drummond J., Hood G.R., Egan S.P. (2019). Cryptic diversity in the North American *Dromochous* tiger beetles (Coleoptera: Carabidae: Cicindelinae): A congruence-based method for species discovery. Zool. J. Linn. Soc..

[14] Duran D.P., Laroche R.A., Gough H.M., Gwiazdowski R.A., Knisley C.B., Herrmann D.P., Roman S.J., Egan S.P. (2020). Geographic Life History Differences Predict Genomic Divergence Better than Mitochondrial Barcodes or Phenotype. Genes.

[15] Erwin T.L., Pearson D.L. (2008). A Treatise on the Western Hemisphere Caraboidea (Coleoptera). Their Classification, Distributions, and Ways of Life.

[16] Rivalier É. (1954). Démembrement du Genre Cicindela Linné, II. Faune Am. Rev. Fr. Entomol..

[17] Rivalier É. (1971). Remarques sur la tribu des Cicindelini (Col. Cicindelidae) et sa subdivision en soustribus. Nouv. Rev. Entomol..

[18] Wiesner J. (1992). Verzeichis der Sandlaufkäfer der Welt.

[19] Gough H.M., Duran D.P., Kawahara A.Y., Toussaint E.F.A. (2018). A comprehensive molecular phylogeny of tiger beetles (Coleoptera, Carabidae, Cicindelinae). Syst. Èntomol..

[20] Dejean P.F.M.A., Aubé C. (1831). Species Général des Coléoptères de la Collection de M. le Comte Dejean.

[21] Normandeau Associates, Inc. (2016). ILP Study 26 Cobblestone and Puritan Tiger Beetle Survey Study Report: In Support of Federal Energy Regulatory Commission Relicensing of: Wilder Hydroelectric Project (FERC Project No. 1892-026) Bellows Fall, Hydroelectric Project (FERC Project No. 1855-045), Vernon Hydroelectric Project (FERC Project No. 1904-073).

[22] Leonard J.G. (1998). Bell RT Northeastern Tiger Beetles. A Field Guide to Tiger Beetles of New England and Eastern Canada.

[23] Hudgins R.M. (2010). Habitat Selection, Dispersal and Detectability of Cobblestone Tiger Beetles (*Cicindela marginipennis* Dejean) along the Genesee River, New York. Master’s Thesis.

[24] USFWS, USA Fish and Wildlife Service (2018). Species Status Assessment (SSA) Report for the Cobblestone Tiger Beetle (Cicindela marginipennis) Version 1.1.

[25] Schlesinger M.D., Novak P.G. (2011). Status and conservation of an imperiled tiger beetle fauna in New York State, USA. J. Insect Conserv..

[26] Federal Register (1984). Review of Invertebrate Wildlife for Listing as Endangered or Threatened Species. Endangered and Threatened Wildlife and Plants.

[27] Federal Register (2019). Twelve Species Not Warranted for Listing as Endangered or Threatened Species. Endangered and Threatened Wildlife and Plants.

[28] Gwiazdowski R. (2020). Report on the First Observation of Mitochondrial Diversity, and Morphological Descriptions of Larvae for the Cobblestone Tiger Beetle.

[29] Ward M.A., Mays J.D. (2010). New Records of The White Mountain Tiger Beetle, *Cicindela ancocisconensis* Harris (1852), and First Record of the Cobblestone Tiger Beetle, *Cicindela marginipennis* Dejean (1831) In *Maine*. Cicindela.

[30] Hebert P.D.N., Penton E.H., Burns J.M., Janzen D.H., Hallwachs W. (2004). Ten species in one: DNA barcoding reveals cryptic species in the neotropical skipper butterfly *Astraptes fulgerator*. Proc. Natl. Acad. Sci. USA.

[31] Hajibabaei M., Janzen D.H., Burns J.M., Hallwachs W., Hebert P.D.N. (2006). DNA barcodes distinguish species of tropical Lepidoptera. Proc. Natl. Acad. Sci. USA.

[32] Simon C., Frati F., Beckenbach A., Crespi B., Liu H., Flook P. (1994). Evolution, Weighting, and Phylogenetic Utility of Mitochondrial Gene Sequences and a Compilation of Conserved Polymerase Chain Reaction Primers. Ann. Èntomol. Soc. Am..

[33] Crozier R.H., Crozier Y.C. (1992). The cytochrome b and ATPase genes of honeybee mitochondrial DNA. Mol. Biol. Evol..

[34] Ratnasingham S., Hebert P.D.N. (2007). BOLD: The Barcode of Life Data System (www.barcodinglife.org). Mol. Ecol. Notes.

[35] Edgar R.C. (2004). MUSCLE: Multiple sequence alignment with high accuracy and high throughput. Nucleic Acids Res..

[36] Templeton A.R., Crandall K.A., Sing C.F. (1992). A Cladistic Analysis of Phenotypic Associations with Haplotypes Inferred from Restriction Endonuclease Mapping and DNA Sequence Data. III. Cladogram Estimation. Genetics.

[37] Clement M., Posada D., Crandall K.A. (2000). TCS: A computer program to estimate gene genealogies. Mol. Ecol..

[38] Huelsenbeck J.P., Ronquist F. (2001). MRBAYES: Bayesian inference of phylogeny. Bioinformatics.

[39] Ronquist F., Teslenko M., van der Mark P., Ayres D.L., Darling A., Höhna S., Larget B., Liu L., Suchard M.A., Huelsenbeck J.P. (2012). MRBAYES 3.2: Efficient Bayesian phylogenetic inference and model selection across a large model space. Syst. Biol..

[40] Miller M., Pfeiffer W., Schwartz T. Creating the CIPRES Science Gateway for Inference of Large Phylogenetic Trees. Proceedings of the 2010 Gateway Computing Environments Workshop (GCE).

[41] Shelford V.E. (1908). Life-histories and larva habitats of the tiger beetles (Cicindelidae). J. Linn. Soc. (London) Zoo.

[42] Gwiazdowski R.A., Gillespie S., Weddle R., Elkinton J.S. (2011). Laboratory Rearing of Common and Endangered Species of North American Tiger Beetles (Coleoptera: Carabidae: Cicindelinae). Ann. Entomol. Soc. Am..

[43] Gwiazdowski R.A. Personal communication.

[44] Knisley C.B., Pearson D.L. (1984). Biosystematics of larval tiger beetles of the Sulphur Springs Valley, Arizona. Trans. Am. Ent. Soc..

[45] Valenti M.A. (1996). Synopsis of reported larval descriptions of tiger beetles (Coleoptera: Cicindelidae) from North America north of Mexico. Cicindela.

[46] Hamilton C.C. (1925). Studies on the morphology, taxonomy, an ecology of the larvae of Holarctic tiger-beetles (Family Cicindelidae). Proc. USNM.

[47] Leffler S.R. (1979). Tiger Beetles of the Pacific Northwest (Coleoptera: Cicindelidae). Ph.D. Thesis.

[48] Willis H.L. (1967). Bionomics and zoogeography of tiger beetles of saline habitats in the central United States. Univ. Kans. Sci. Bull..

[49] Gaumer G.C. (1977). The Variation and Taxonomy of *Cicindela formosa* Say (Coleoptera: Cicindelidae). Ph.D. Thesis.

[50] Horn G.H. (1878). Descriptions of the Larvae of North American Genera of Cicindelidae, also of Dicaelus with a Note on Rhynchophorus. Trans. Am. Ent. Soc..

[51] Beatty D.R., Knisley C.B. (1982). A description of the larval stages of *Cicindela rufiventris* Dejean (Coleoptera:Cicindelidae). Cicindela.

[52] Kaulbers M.M., Freitag R. (1993). A description of the third instar larva of *Cicindela denikei*. Cicindela.

[53] Spanton T.G. (1988). The *Cicindela sylvatica* group: Geographical variation and classification of the neartic taxa, and constructed phylogeny and geographic history of the species. Quaest. Entomol..

[54] Willis H.L. (1980). Description of the larva of *Cicindela patruela*. Cicindela.

[55] Knisley C.B., Schultz T.D. (1997). The Biology of Tiger Beetles and a Guide to the Species of the South Atlantic States.

[56] Gwiazdowski R.A. Personal communication.

[57] Pineda P.M., Kondratieff B.C. (2002). The Larvae of *Cicindela theatina* (Coleoptera: Cicindelidae); A Regional North American Sand Dune Endemic. Entomol. News.

[58] Barraclough T.G., Vogler A.P. (2002). Recent Diversification Rates in North American Tiger Beetles Estimated from a Dated mtDNA Phylogenetic Tree. Mol. Biol. Evol..

[59] Duran D.P. (2010). Speciation and Diversification in The North American Tiger Beetles Of The *Cicindela sylvatica* Group: Morphological Variation and an Ecophylogeographic Approach. Ph.D. Thesis.

[60] Diogo A., Vogler A., Gimenez A., Gallego D., Galián J. (1999). Conservation Genetics of Cicindela Deserticoloides, an Endangered Tiger Beetle Endemic to Southeastern Spain. J. Insect Conserv..

[61] Doorenweerd C., Jose M.S., Barr N., Leblanc L., Rubinoff D. (2020). Highly Variable COI Haplotype Diversity between Three Species of Invasive Pest Fruit Fly Reflects Remarkably Incongruent Demographic Histories. Sci. Rep..

[62] Gwiazdowski R.A., Elkinton J., Sremac M., Dewaard J.R. (2013). Phylogeographic Diversity of the Winter Moths Operophtera brumata and *O. bruceata* (Lepidoptera: Geometridae) in Europe and North America. Ann. Èntomol. Soc. Am..

[63] Luo Y., Agnarsson I. (2018). Global MtDNA Genetic Structure and Hypothesized Invasion History of a Major Pest of Citrus, *Diaphorina citri* (Hemiptera: Liviidae). Ecol. Evol..

[64] Knisley C.B., Gowan C., Wirth C. (2018). Effects of soil moisture, vegetation and food on adult activity, oviposition and larval development in the tiger beetle, *Cicindela albissima* Rumpp. J. Insect Conserv..

[65] Gwiazdowski R.A. (2020). Interim Report on observed natural oviposition for the Puritan tiger beetle, Ellipsoptera (Cicindela) puritana, in southern Connecticut. Unpublished report to the USA Fish and Wildlife Service.

[66] Cunningham A.A., Daszak P. (1998). Extinction of a Species of Land Snail Due to Infection with a Microsporidian Parasite. Conserv. Biol..

[67] Goldstein P.Z. (2018). Personal Communication.

[68] Cornelisse T.M., Vasey M.C., Holl K.D., Letourneau D.K. (2012). Artificial bare patches increase habitat for the endangered Ohlone tiger beetle (*Cicindela ohlone*). J. Insect Conserv..

[69] Gwiazdowski R.A. (2019). Puritan Tiger Beetle (PTB) Recovery Project 2018 Annual Report.

[70] Dunn G.A., Wilson D. (1979). *Cicindela marginipennis* in New Hampshire. Cicindela.

[71] Nothnagle P. (1995). Survey of the White River for the Cobblestone Tiger Beetle, *Cicindela marginipennis*.

[72] Kritsky G., Cortright B., Duennes M., Smith J., Pierce S. (2009). The Status of *Cicindela marginipennis* (Coleoptera: Carabidae) In Southeastern Indiana. Proc. Indiana Acad. Sci..

[73] Hudgins R., Norment C., Schlesinger M.D., Novak P.G. (2011). Habitat Selection and Dispersal of the Cobblestone Tiger Beetle (*Cicindela marginipennis* Dejean) along the Genesee River, New York. Am. Midl. Nat..

[74] Hudgins R.M., Norment C., Schlesinger M.D. (2011). Assessing detectability for monitoring of rare species: A case study of the cobblestone tiger beetle (*Cicindela marginipennis* Dejean). J. Insect Conserv..

[75] Mawdsley J.R. (2007). Use of simple remote sensing tools to expedite surveys for rare tiger beetles (Insecta: Coleoptera: Cicindelidae). J. Insect Conserv..

